# Identification of Skin Electrical Injury Using Infrared Imaging: A Possible Complementary Tool for Histological Examination

**DOI:** 10.1371/journal.pone.0170844

**Published:** 2017-01-24

**Authors:** Ji Zhang, Wei Lin, Hancheng Lin, Zhenyuan Wang, Hongmei Dong

**Affiliations:** 1 Department of Forensic Pathology, College of Forensic Medicine, Xi’an Jiaotong University, Xi’an, Shaanxi Province, China; 2 Department of Forensic Medicine, Tongji Medical College, Huazhong University of Science and Technology, Wuhan, Hubei Province, China; University of Queensland Diamantina Institute, AUSTRALIA

## Abstract

In forensic practice, determination of electrocution as a cause of death usually depends on the conventional histological examination of electrical mark in the body skin, but the limitation of this method includes subjective bias by different forensic pathologists, especially for identifying suspicious electrical mark. The aim of our work is to introduce Fourier transform infrared (FTIR) spectroscopy in combination with chemometrics as a complementary tool for providing an relatively objective diagnosis. The results of principle component analysis (PCA) showed that there were significant differences of protein structural profile between electrical mark and normal skin in terms of α-helix, antiparallel β-sheet and β-sheet content. Then a partial least square (PLS) model was established based on this spectral dataset and used to discriminate electrical mark from normal skin areas in independent tissue sections as revealed by color-coded digital maps, making the visualization of electrical injury more intuitively. Our pilot study demonstrates the potential of FTIR spectroscopy as a complementary tool for diagnosis of electrical mark.

## Introduction

One of the most important aspects in forensic science is electrocution diagnosis. As electrical fatalities are generally caused by cardiac arrhythmia, respiratory arrest and damage to brainstem, autopsy findings are not evident or non-specific in the multiple vital organs [1, 2]. Electrical mark reflects the fact that the skin contacts with electrical conductors. Its pathological examination is crucial for diagnosis of fatal electrocution, especially when the death circumstances are suspicious [3, 4]. Histologically, it is well-known that epidermal nuclear elongation is a typical hallmark of electrical mark and of great diagnostic value for electrocution. However, histological examination is primarily dependent upon the individual subjective judgement, and thus forensic pathologists usually incorporate other methods with routine histopathology for determination of this mark[5], such as computerized image analysis[6] and detection of metallization by scanning electron microscopy[7]. But few provide more insights into the electrical mark at the molecular level, and make an objective diagnosis.

Typical imaging methods such as confocal laser microscopy and multiphoton microscopy have been instrumental to exploit certain structures and molecules of the skin without the need for tissue processing[8–10]. Despite their high sensitivity and spatial resolution, it is almost impossible with them to make a globally chemical analysis in biological tissues. Vibrational spectroscopy is a bio-analytical tool capable of detecting component and structural alterations that occur in molecules within samples according to their chemical bonds. The complimentary techniques mainly include Raman and Fourier transform infrared (FTIR) spectroscopy. Since various macromolecules in biological tissues have their specific absorption bands in an infrared spectrum, the use of vibrational spectroscopy as a valuable tool is becoming widespread in biomedicine. Combination of this spectroscopic method with an infrared microscopy achieves stain free biochemical imaging and chemical analysis in a small area of tissue sections, thus providing an ideal platform by which diseased or abnormal areas can be investigated at the molecular level. Using chemometrics, multiple histological classes on the spectroscopic image can be identified and visualized as a digitally classified image[11, 12].

To the best of our knowledge, few attempts to characterize electrical mark using vibrational spectroscopy has been made[13], especially in combination with chemometrics for automatic diagnosis. In contrast to Raman spectroscopy, FTIR spectroscopy appears to provide more bioinformation with higher signal-noise ratio. Therefore, we performed a FTIR investigation on paraffin sections of human hands by principle component analysis (PCA) in order to determine variance of protein structures between electrical mark and normal skin. Subsequently, a partial least square (PLS) model based on the acquired spectral data was established to identify the electrical mark intuitively in the form of spectral pseudo color image.

## Materials and Methods

### Sample collection and preparation

The work involving the use of human specimens was performed after informed written consents were obtained from family members of the victims. The ethics committee of Xi’an Jiaotong University specifically approved this study. Skin samples were collected from 23 autoptic electrocution cases admitted to the college of forensic medicine at Xi’an Jiaotong University. In these cases, the corpses were stored at 4°C within 24h postmortem. All the victims died in their own home, and the hand electrical marks were caused by domestic power supply of China at a voltage of 220 volts. In the control group, the normal skins were sampled at the contralateral hand from the same corpses.

Upon fixing with 4% buffered paraformaldehyde for 24h, the skin specimens were embedded in paraffin and cut into 6 μm-thick slices by a microtome. The sections were mounted on 3 mm-thick infrared transparent slides for FTIR analysis, which were made by the barium fluoride (BaF_2_), (Beijing optical instrument factory, Beijing, China) and then dewaxed and dehydrated with xylene and ethanol respectively. Adjacent sections at the same samples were mounted on standard glass slides for histological examination. In this study, the skin samples in 16 cases were used as the calibration group while those in the remaining 7 cases served as the validation group.

### FTIR data collection and pre-processing

FTIR spectra within the mid-IR region of 900–4000 cm^-1^ were acquired in transmission mode using an FTIR spectrometer (Thermo Scientific Nicolet TM 5700-II, MIT, USA) coupled to an infrared microscope (Nicolet Continue μmXL, MIT, USA), equipped with liquid nitrogen cooled Mercury Cadmium Telluride (MCT) high sensitive detection and a high-intensity long-lasting ETC EverGlo^※^mid-IR source. An area of 140 × 200 μm^2^ on a tissue section was scanned with microscopic aperture of 40×40 μm^2^ at a step of 10 μm, where each spectrum was obtained at a resolution of 16 cm^-1^ and 8 scans. To get rid of the interference of atmospheric water vapor, background spectra were collected on the blank area of BaF_2_ slides and then automatically subtracted from each spectrum under a relative humidity of 20%.

FTIR spectral collection was carried out using the OMNIC Picta 8.0 (Thermo Fisher, U.S.A) and data pre-processing was performed with the Matlab 2014a (MathWorks, U.S.A) equipped with PLS Toolbox 8.1.1 (Eigenvector Research, Inc.). The raw spectra were normalized by extended multiplicative signal correction (EMSC) and then transformed to the second derivatives using a Savitzky-Golay smoothing function (9 points) in order to minimize baseline offset and the difference of thickness on the tissue sections. FTIR imaging on the tissue section was constructed using “jet” color scheme, showing the intensity distribution of peak absorbance from different protein secondary structures as reflected in the Amide I band (1710–1585 cm^-1^) in a spectrum. The red color indicated the highest relative concentration while blue color represented the lowest intensity.

### Multivariable statistical analysis

Multivariable statistical analysis was also conducted using the Matlab 2014a (MathWorks, U.S.A) equipped with PLS Toolbox 8.1.1 (Eigenvector Research, Inc.). Our investigation was primarily focused on the spectral range from 1710 to 1585 cm^-1^ as it was strongly associated with protein conformation in biological tissues.

PCA was employed to interpret the covariance structure of data using a small number of components which are linear combination of original variables[14]. Specifically, PCA extracts a set of correlated variables and then converts them into a smaller set of uncorrelated variables named as principle components (PCs), while maintaining as much of the information in the original data as possible. The classification result is expressed by the projection of the samples on a set of orthogonal PCs, and the contribution of variables into the classification can be evaluated by loading plots associated with corresponding PCs. In the calibration group, 35 spectra were obtained randomly within the areas of electrical mark while 46 spectra were acquired within the normal epidermal areas. These spectral dataset were pre-processed as described above. Leave-one-out cross-validation (LOOCV) was performed, and eight PCs were selected for initial PCA decomposition. Two dimensional score plot along two principle components (PCs), and corresponding loading plot were graphed in order to identify the potential spectral features for discrimination between normal epidermis and electrical mark.

Unlike PCA, PLS model can extract simultaneously components called as latent factors from the predictor variables X and the response variables Y for constructing a predictive model. In this study, the spectra from the areas of electrical mark (35 spectra), normal dermis (27 spectra) and epidermis (34 spectra) were collected respectively in the calibration group for PLS modelling. The predictor variables X corresponded to the matrix of spectral intensity while the response variables Y were represented by the dummy variables of 1, 2 and 3 for normal epidermis, electrical mark and normal dermis respectively. LOOCV was performed to evaluate the classification among groups as revealed in PLS score plot. In order to provide more accurate prediction, it is essential to determine the Y value range of different groups and evaluate the performance of the developed model. Therefore, an internal predication was performed by the initial PLS model in an additional dataset including 48 spectra from the three areas, all of which were also collected in the calibration group but not included for PLS modelling. In the external validation, spectra were collected within the areas of electrical epidermis, normal epidermis and normal dermis of the unknown tissue sections (the validation group) respectively. Based on the acquired thresholds of Y value, these spectra were classified by the trained PLS model in a single-blinded manner, whose identity was not available for the analyst.

## Results and Discussion

**Fig 1:**
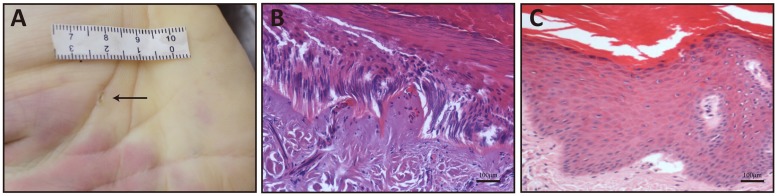
Macroscopic and microscopic images of electrical mark and normal skin. (A) macroscopic features of electrical mark in the hand. Microscopic features of electrical mark (B) and normal skin (C).

The skin samples in all cases were first subjected to morphological examination to determine the area of electrical mark for FTIR analysis. As exemplified in Fig 1, macroscopic examination showed hallmarks of the electrical injury characterized by pale, cave-like lesions with a raised margin. Microscopically, electrical mark revealed separation of epidermis and dermis, elongated epidermal cells arranged in a polarized direction and darkly stained cell nuclei in contrast to normal skin.

**Fig 2:**
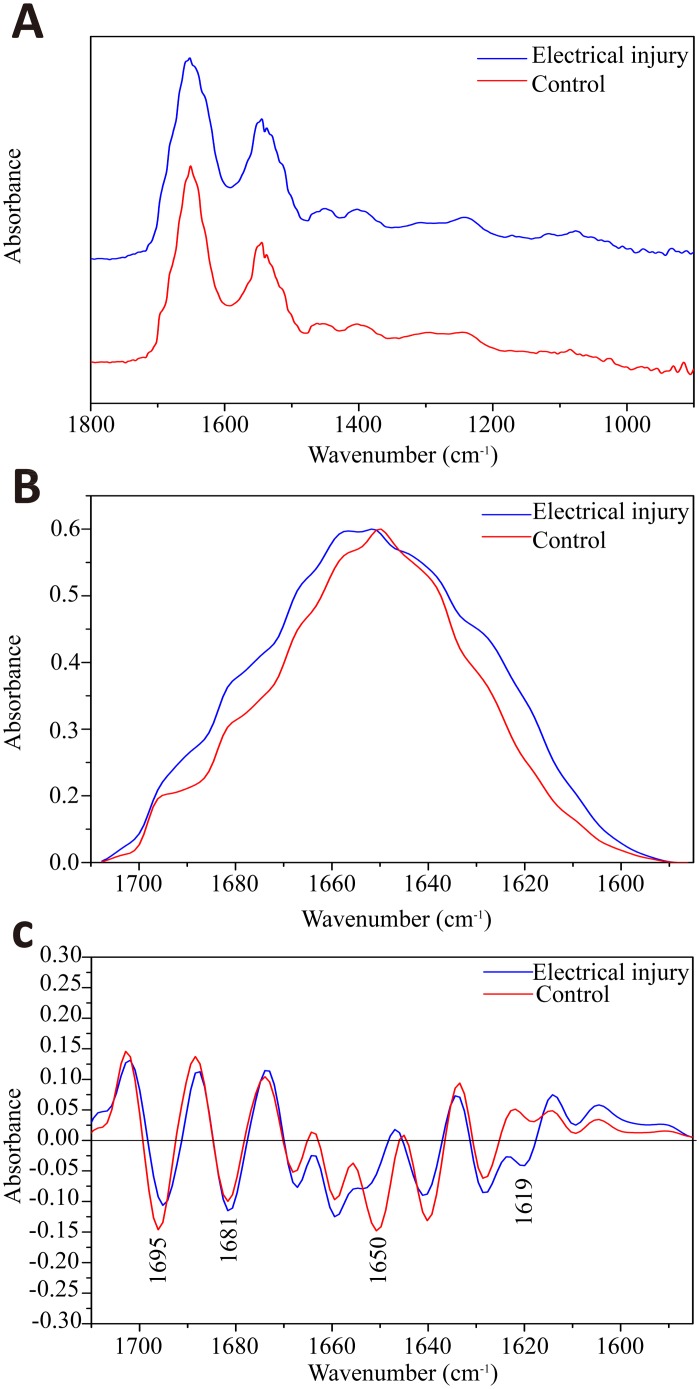
A comparison of absorbance and second derivative spectra between electrical mark and normal skin. (A) absorbance spectra within 1800–900 cm^-1^ for both categories. Superimposition of them within the Amide I region in absorbance mode (B) and second derivative mode (C). The blue and red spectra represent electrical injury and normal epidermis respectively.

Compared with histological examination, infrared spectra were obtained from the areas of normal epidermis and electrical mark (the area of epidermal cell elongation) respectively in serial unstained sections. Since organic solvents have great impact on lipids and carbohydrates of biological tissues, our investigation was focused on protein changes on the skin sections. Fig 2 shows a comparison of the average normalized absorbance and second derivative spectra from the electrical mark and normal epidermis. On the first inspection, the maximal band which is referred to as Amide I band were generally identical in the absorbance spectra of the two groups. This band mainly originates from the C = O stretching with little contribution from the N-H bending of proteins (Fig 2A), and is particularly sensitive to alterations in protein conformation in terms of their maximum wavenumber as well as peak shape [15–17]. As seen in Fig 2B, the Amide I band for normal epidermis is centered at 1650 cm^-1^ whereas electrical injury group is shifted to the higher wavenumber with a wider band width. The peak deviation and deformation suggest that electricity causes distinct changes in protein structures of the skin. Second derivative transformation is a common method of resolving overlapping bands, where the negative peaks are directly aligned to the center peak of non-derivative spectrum. In this study, this pre-processing method was used to identify several peaks from C = O stretching of various protein structures, which were included within the Amide I region[18–20]. Fig 2C illustrates superimposition of second derivative spectra between control and electrical mark groups. The peaks around 1650 cm^-1^ (α-helix) and 1695cm^-1^ (antiparallel β-sheet) contributed greatly to the spectral hallmark of normal epidermis. In contrast, epidermal electrical mark was characterized by higher intensities of β-sheet (1619cm^-1^) and β-turn (1681cm^-1^).

**Fig 3:**
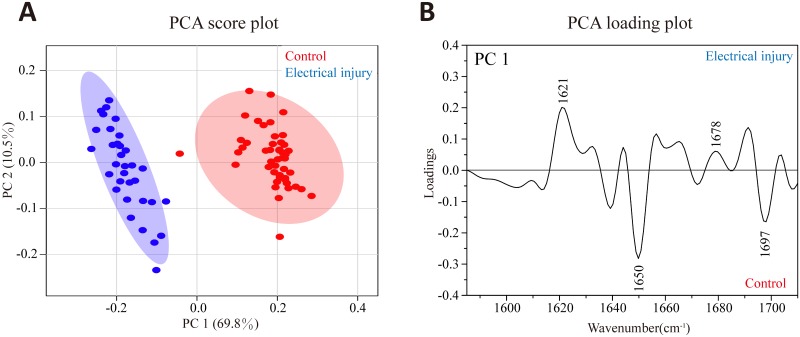
The results of PCA based on the Amide I band. (A) the score plot along PC 1 versus PC 2 is obtained where the blue and red circles represent the spectra from electrical injury and normal epidermis (control) respectively, and the larger circle is 95% confidence interval. (B) PCA loading plot associated with PC 1 is obtained where the negative peaks are characteristic for normal epidermis (control) and positive for electrical injury.

To further visualize the subtle changes in protein secondary structures, PCA was applied to second derivative spectra within the Amide I region. The score plot (shown in Fig 3A) demonstrates a complete discrimination of scattering points between electrical mark and control groups along the PC 1 which accounted for 69.8% of total variation. This suggests that most spectral variances were associated with changes in protein conformation of the electrical mark, and PC 1 extracted from the original dataset is mainly responsible for the distinction. Therefore, PC 1-related loading plot as shown in Fig 3B was used to identify spectral features for electrical mark. Consistent with the second derivative spectra, this loading plot shows that the discriminating negatively correlated loadings were found for normal epidermis at 1650 and 1697 cm^-1^ whereas positive loadings representative for electrical injury were present at 1621 and 1678 cm^-1^. The observed spectral pattern is similar to those of bovine serum albumin (BSA) with increasing temperatures, which reveals that gradually decreased α-helix content with predominance of β-sheet as the temperature increases [21]. Therefore, this result may be associated with protein denaturation caused by Joule heating. Indeed, several studies preferably suppose that epidermal cell elongation mainly results from mechanical compression due to swollen dermis rather than protein conformation changes even though it is one of the most important pathophysiology of electrical injury[22–24]. Therefore, the contribution of protein conformation changes into the formation of electrical mark remains to be further confirmed. Nevertheless, our study demonstrates the ability of FTIR spectroscopy to identify protein structural changes in electrical mark, further reflecting its high sensitivity to subtle molecular changes in the skin.

**Fig 4:**
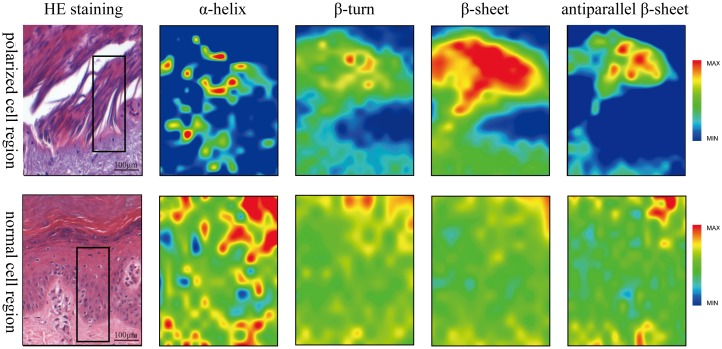
The comparison of FTIR mappings based on protein conformations between electrical mark and normal skin. The first and second rows represent FTIR mappings in the areas of elongation cells and corresponding normal epidermis respectively, in which the peak absorbance intensities associated α-helix, β-turn, β-sheet and antiparallel β-sheet are revealed by “jet” colors with red indicating the highest relative concentration, and blue, the lowest.

The next step of our study was to address the distribution of protein secondary structures in the unstained tissue sections. False color images of normal epidermis and electrical mark were obtained according to their absorbance peak intensity as seen in Fig 4. With respect to the normal epidermis, a higher content of β-sheet was evident but with lower α-helix and anti-parallel β-sheet structures for electrical mark. There was no significant differences in the peak intensity of β-turn content between electrical mark and normal epidermis, which could be interpreted by its little contribution into their separation as revealed by the lower loading intensity of PC 1. Based on these results, it is inferred that some proteins rich in α-helix, anti-parallel β-sheet and β-sheet may serve as biomarkers for identification of electrical mark.

**Fig 5:**
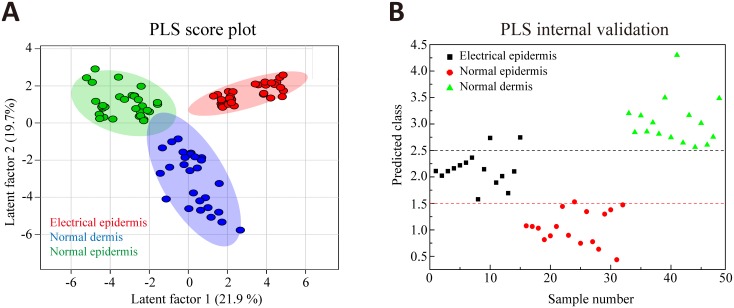
The results of PLS classification based on spectral dataset in the calibration. (A) PLS score plot shows that all groups representing different structures of the skin are well-separated along latent factor 1. (B) Prediction result in an additional dataset that are not included for PLS modelling shows that electrical epidermis, normal epidermis and dermis can be distinguishable from each other by using the PLS model. Red and black dot lines show classification threshold for separating the three categories respectively.

**Fig 6:**
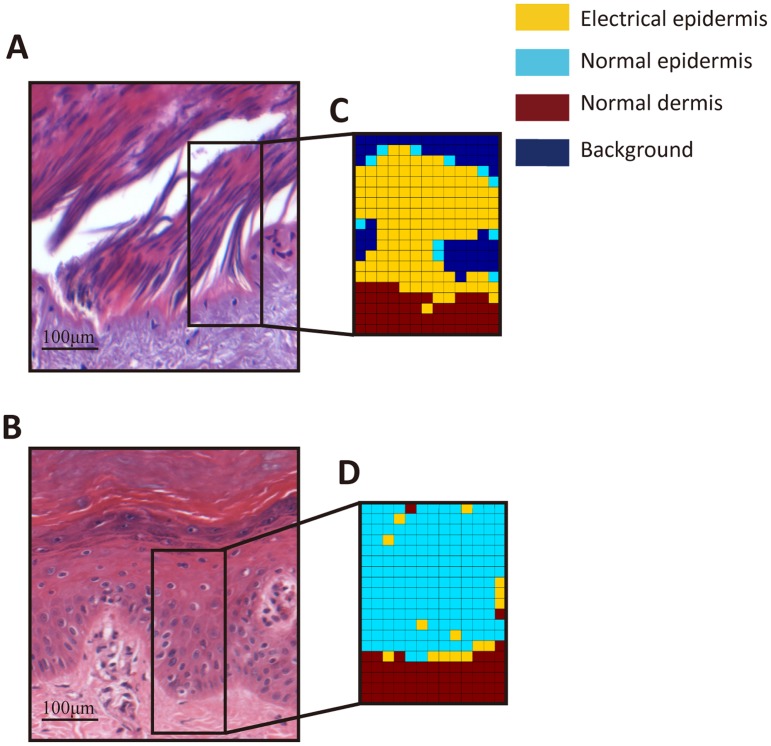
The identification of electrical mark by the FTIR-based PLS model in an independent tissue section. HE stained skin tissue sections are presented here where elongation cell in electrical mark and normal skin regions are included in A and B respectively. The spectra within the black box of the HE staining images were classified by the PLS model, and the prediction results in the electrical mark (C) and normal skin areas (D) were revealed by pseudo-colored images. Electrical epidermis, normal epidermis and normal dermis appear in yellow, light blue and brown respectively.

Although infrared imaging based on absorbance intensity appears to be an alternative method to detect electrical mark, it is still required to discriminate and process FTIR spectra by skilled personnel. Therefore, this promote us to develop a FTIR-based method for auxiliary diagnosis in cases of electrocution, which could provide a more objective and intuitive identification of electrical mark than routine histological examination. In this study, the PLS model was chosen to process the spectral dataset because it has been demonstrated that this supervised pattern recognition method is powerful for distinguishing different sample categories[25–28]. The spectra in the calibration group were used to establish the initial PLS model. The LOOCV result as shown in Fig 5A shows that scattering points are clustered based on their groups, and distinguishable from each other along latent factor 1 which explains 21.9% of total variances. This suggests detectable differences of protein compositions among them. Subsequently, the initial PLS model performed an prediction test in an additional dataset which were also collected in the calibration group but not included for model calibration. As seen from Fig 5B, both the distinct separation among the three classes and correct classification are generally visible, suggesting that the established PLS model has the ability to discriminate different skin structures. The range of Y values for more efficient distinction among the classes were determined (0–1.5 for normal epidermis, 1.5–2.5 for electrical injury and greater than 2.5 for normal dermis), where most of score points representing the spectra from different classes were within their own assigned Y ranges. In the external validation, 17, 20 and 14 spectra were selected within the areas of electrical epidermis, normal epidermis and normal dermis in the validation group respectively. In Table 1, the trained PLS model shows a satisfactory prediction in these spectral dataset, and the accuracy of electrical and normal epidermis is up to 85%. Fig 6 displays two spectral mappings from normal skin and electrical mark in a typical case of the validation group, each of which were composed of 280 spectra (14×20 matrix), and their assignments were predicted by the PLS model. Different groups were discriminated by colors in a color-coded digital map (yellow: electrical mark; light blue: normal epidermis; brown: normal dermis; dark blue: background). As illustrated in Fig 6, the areas classified are globally co-localized with different structures of the skin, even though little spectra are erroneously assigned.

**Table 1:** The result of prediction in the validation group by PLS model.

Class	The range of predicted Y	Correct	False	Accuracy
Normal epidermis	0–1.5	17/20	3/20	85.0%
Electrical epidermis	1.5–2.5	15/17	2/17	88.2%
Normal dermis	>2.5	11/14	3/14	78.6%

In forensic practice, the histological examination of electrical mark in the skin is of great diagnostic value in electrocution cases. However, determination of such an electrical injury is primarily dependent upon subjective judgement by experienced forensic pathologist, and thus it is sometimes difficult to draw a conclusion when a suspicious electrical mark is present. Therefore, FTIR-based mathematical models may be an ideal candidate as supplements to histological examination. Firstly, this technique has the ability to achieve chemical analysis at the molecular level which may be more sensitive to subtle biochemical changes than optical observation. Secondly, spectral information on electrical mark can be learned by pattern recognition techniques ahead, and then they are used to make an relatively objective decision in an independent tissue section. Thirdly, there is non-interference between FTIR analysis and histological examination due to non-destructive nature of FTIR analysis.

## Conclusions

In our study, FTIR spectroscopy with an infrared microscopy was applied to characterize protein conformations of electrical mark in the human skin. The observed spectral variances between electrical mark and normal skin suggest that some skin proteins rich in α-helix, antiparallel β-sheet and β-sheet may be used as biomarkers for identification of electrical mark. Further, the application of FTIR imaging in combination with PLS model appeared to make a more objective and intuitive diagnosis than routine histological examination.

Nevertheless, it doesn’t mean that the approach used here is much better than traditional histological methods because much work should be done before application of this method in real practice. For example, it is also important to find out differences between electrical marks and similar morphological changes such as warts, callous thickening of the epidermis, thermal injury[29] and abrasion[5]. The purpose of our study is to develop a complementary tool along with traditional methods for providing a more accurate diagnosis. In the future, advanced FTIR instruments equipped with more intense light source (i.e. synchrotron infrared light source) and sensitive detector (i.e. focal plane array detector) are required as they can provide higher spatial resolution and faster data collection.

## Abbreviations

BSA: Bovine serum albumin
EMSC: Extended multiplicative signal correction
FTIR: Fourier transform infrared
LOOCV: Leave-one-out cross-validation
MCT: Mercury Cadmium Telluride
PCA: Principle component analysis
PCs: Principle components
PLS: Partial least square
